# The democratization of scientific publishing

**DOI:** 10.1186/s12916-019-1249-1

**Published:** 2019-01-18

**Authors:** Clare Fiala, Eleftherios P. Diamandis

**Affiliations:** 1 Department of Pathology and Laboratory Medicine, Mount Sinai Hospital, Toronto, ON Canada; 2 Department of Laboratory Medicine and Pathobiology, University of Toronto, Toronto, ON Canada; 3 Department of Clinical Biochemistry, University Health Network, Toronto, ON Canada; 4 FRSC Head of Clinical Biochemistry, Mount Sinai Hospital and University Health Network, 60 Murray St., Floor 6, Rm L6-201, Box 32, Toronto, ON M5T 3L9 Canada

**Keywords:** Scientific publishing, Impact factor, Open access, Anxiety, Depression, Young scientists

## Abstract

Where should I submit my paper? This is a question that young scientists and trainees frequently ask. In this Commentary, we advise on how to make such a decision whilst balancing the risks and benefits. We argue that trying to publish in top tier journals may not always be the best option and that publishing in indexed, open access journals may expose research to the same or larger audiences. The value of research should not be judged according to the publishing journal’s name, but rather from other measures of impact such as successful commercialization of new technologies, number of citations, and downloads. We also highlight the role of mentors, who have the responsibility to protect the long-term interests of their trainees by balancing the consequences of acceptances and rejections.

## Introduction

One of the most frequent dilemmas we face as supervisors is where to submit a paper prepared by our trainees. Typically, we ask students to prepare a list of possible journals, along with their respective journal impact factors (JIFs). After editing the work, a discussion takes place with the trainee about where to submit the manuscript. Expectedly, the majority of students wish to publish their papers to the highest possible impact journals, as judged by the JIF. Herein, we discuss the implications of trying to publish in elite journals and the consequences of this on the career and wellbeing of young investigators.

Numerous investigators maintain personal subscriptions to *Nature* and *Science* magazines. In our view, the value of these journals is mostly related to their high-quality editorial content, with very few papers published in these multidisciplinary journals being directly related to our work (the discovery and validation of novel cancer biomarkers). Indeed, for our research purposes, we retrieve most papers by either searching PubMed, or through alerts, based on keywords. We assume that most scientists, young and old, follow similar strategies.

Since the 1970s, scientific publishing has changed dramatically (for a recent review of these changes please see our previous publication [1]); we used to spend considerable time in the library, which is now a thing of the past, and we used to photocopy reams of papers, whereas now we photocopy none. Most importantly, the invention of the Internet ushered in online, open access journals, providing readily available papers, in full text, without the need for a subscription.

We have always advised our graduate students to read widely. In our lab, weekly meetings have become more diverse, with our ‘news and views’ section now covering areas mostly unrelated to our core research interests of translational medicine and cancer biomarkers. For example, in one of our latest journal clubs, our students presented efforts to avoid the extinction of the white rhinoceros, how to use social media to avoid suicide attempts, and some recent developments in the technology and ethics of autonomous cars.

### Where to submit?

We follow at least three rules for choosing where to submit our work, and generally favor quick publishing and/or open access platforms. First, we make sure the journal is indexed in PubMed, so that it can be retrieved by searching; secondly, we identify and avoid ‘predatory’ journals – sham journals that profit by charging unsuspecting contributors fake ‘author fees’ [2]; and thirdly we ensure the journal is affiliated with a credible, even if small, publisher or a recognized professional association.

We agree with the notion that publishing a paper in a top tier journal may help secure better employment as well as help in being awarded major grants or prizes, among other benefits. However, these journals have some inherent issues. We recently argued that irreproducibility of papers in high-impact journals may be more frequent, and more dangerous (termed as ‘malignant’), than in lower impact journals (termed as ‘benign’) [3–5]. We and others have also repeatedly argued that the publication of a paper in a prestigious journal should not be used to extrapolate that the paper itself is valuable, impactful, or of high quality [6, 7]. We consider that other measures of impact, such as citations, patents, startup companies, and development of consumer products, may be of greater importance. Consequently, we advise our trainees to submit their papers to prestigious journals, if they so wish, but to be emotionally prepared to accept rejections, in many cases without their paper even being reviewed.

Young investigators may be prefixed with the JIF. However, we recently predicted the future demise of the JIF and, indeed, we have seen our prediction come true with certain journals [6, 7]. To date, an increasing number of journals prefer not to advertise, or even seek to obtain, their JIF [8]. Furthermore, we recently proposed a new factor, called the CAPCI (citation average per citable item), to avoid using the misleading word ‘impact’ [8], as also proposed by others [9–13].

### High-impact hazards

Not many senior researchers discuss the fact that submitting to the highest-impact journals may be associated with adverse effects, especially for their trainees. It is now common knowledge that anxiety and depression among graduate students, post-docs, and young faculty is on the rise, with the issue attracting significant attention [14–16]. One reason, among many, is the tremendous competition between young investigators for jobs, publications, awards, grants, etc. Rejection of papers in high-impact journals is, understandably, painful and damaging to the morale and self-esteem of early career researchers; one of us (EPD) has witnessed these adversities very frequently over 30 years. Repeated cycles of rejections could inflict depression, anxiety, and other mental illnesses, or even suicidal thoughts [16–18].

### Open access journals

In our view, the importance of publishing in high-impact journals (many of which are closed access, and therefore subscription journals) is progressively decreasing due to the rise of open access journals and other publication options. In open access journals, upon publication, research is immediately visible in full text to international audiences who can read and cite it. It is difficult to envisage that any researcher would decide whether or not to read a highly relevant paper for their research based on the journal’s rating by the JIF (unless, of course, the journal is categorized as ‘predatory’). A recent analysis demonstrated that the number of citations accrued by a paper is not correlated with the JIF if the journal is open access [19]. Consequently, there is no reason why a paper should lose any citations or audience attention due to being published in an indexed, open access journal.

### Preprint servers vs. journals

There are also alternative methods for the publication of research, such as submission to a pre-print server. Pre-print servers are repositories that archive papers and make them available to readers without peer review. The major advantages of such a practice are the immediate visibility and the establishment of some priority in a specific subject. Thus, possible delays with the review process (which sometimes extend to a year or more) are avoided. Our students are increasingly advised to submit to such pre-print servers, yet they are suspicious that somebody may steal their ideas prior to formal acceptance by a peer-reviewed journal. The disadvantages of pre-print servers are that you may lose the benefit of peer review to improve your paper before it becomes public and that some journals have strict qualification rules regarding the prior submission to a pre-print server.

We are now increasingly advising our young associates to consider indexed, open access, quick publication journals. While practices vary, some of these journals have additional unique features besides being open access. For instance, they will accept and post a paper on their website (but not in PubMed) prior to formal acceptance by peer review, the reviewers are identifiable, and the reviews are public, and/or they require the submission of complete primary data to allow reanalysis by others. Following acceptance, the paper is freely available in full text in PubMed. Our experience is that, despite some concerns about the JIF, which may not even be used by these journals or could be relatively low, our trainees are thrilled to see their paper in PubMed, sometimes within 2–4 months from submission. Such fast visibility is a highly effective anti-anxiety and anti-depressive remedy. Nevertheless, our trainees realize that, under this publication model, their paper will be under permanent review, since readers can post comments at any time, even years after publication. A further perceived disadvantage would be the nominal fee paid by the authors to cover production costs; yet, due to competition, this fee is steadily decreasing. However, authors must realize that there are costs involved in the publication of a paper, even in open access, and that these costs should be met by authors, institutions, or funders. Finally, we also tell our trainees that no Nobel committee will deny them the Prize due to their work having been published in an open access journal without or with a low impact factor.

### Closed access attack

The open access publishing model has attracted increased attention in recent times following the demand of some scientific opinion leaders that all papers published in closed access journals become freely available in open access format, quickly after publication, without cost to the authors. One concern is that closed access journals could impede scientific progress due to their usually slower publication schedules and paywalls; this could be particularly damaging for very fast-growing disciplines such as artificial intelligence. Some authors are even threatening to boycott some highly visible journals if they insist on closed access or charging publication fees in the open access model [20]. Recently, two of the world’s largest biomedical research funders – Wellcome Trust and the Gates Foundation – joined another 13 funders in backing a plan to make all papers resulting from work they fund open access on publication by 2020 [21].

### Role of mentors

We know of many mentors who insist on either publishing in top-tier journals or not publishing at all; their behavior is reminiscent of the Mercedes-Benz car TV advertisement: “*the best or nothing*”. While many mentors can afford this luxury, their trainees likely cannot. Our own experience suggests that, for most students, it is better to publish one or more papers in open access journals, as outlined above, and then proceed to completion of their degrees, on time. The alternative insistence on publishing in the highest impact journals runs the risk of multiple rejections, major delays, years of additional research, and postponement of personal life decisions such as starting a family or finding a job. By guiding their trainees to publish in non-elite, yet respected journals, mentors may prevent serious consequences such as anxiety and related disorders.

## Conclusions

In our view, ‘elite’ journals are still required, yet their value lies on their exemplary editorial content, rather than on their original scientific material. Their science could be published elsewhere, and have the same societal impact and benefit. For example, one of the most influential papers in history is the one-page *Nature* report by Watson and Crick on the double helical structure of DNA [22]. Would the impact of this, or similar papers, be different had they been published in other, low impact factor, indexed journals? We believe not.

For mentors, we advise the adoption of a motto different from that of the Mercedes-Benz car manufacturer and to equip their trainees with the knowledge to make an informed decision as to where to send their work. After all, it is preferable that their hard work is made known to the international community through an open access journal or (bio-)archive than to remain unpublished.
